# High Temperature Near-Field NanoThermoMechanical Rectification

**DOI:** 10.1038/srep44901

**Published:** 2017-03-21

**Authors:** Mahmoud Elzouka, Sidy Ndao

**Affiliations:** 1 Department of Mechanical & Materials Engineering, University of Nebraska-Lincoln, Lincoln, Nebraska 68588, United States

## Abstract

Limited performance and reliability of electronic devices at extreme temperatures, intensive electromagnetic fields, and radiation found in space exploration missions (i.e., Venus & Jupiter planetary exploration, and heliophysics missions) and earth-based applications requires the development of alternative computing technologies. In the pursuit of alternative technologies, research efforts have looked into developing thermal memory and logic devices that use heat instead of electricity to perform computations. However, most of the proposed technologies operate at room or cryogenic temperatures, due to their dependence on material’s temperature-dependent properties. Here in this research, we show experimentally—for the first time—the use of near-field thermal radiation (NFTR) to achieve thermal rectification at high temperatures, which can be used to build high-temperature thermal diodes for performing logic operations in harsh environments. We achieved rectification through the coupling between NFTR and the size of a micro/nano gap separating two terminals, engineered to be a function of heat flow direction. We fabricated and tested a proof-of-concept NanoThermoMechanical device that has shown a maximum rectification of 10.9% at terminals’ temperatures of 375 and 530 K. Experimentally, we operated the microdevice in temperatures as high as about 600 K, demonstrating this technology’s suitability to operate at high temperatures.

Thermal logic devices^1^ can be alternatives to electronic ones, which typically fail in special applications that require high temperatures and ionizing radiation tolerance (e.g., deep-Earth exploration for petroleum and geothermal energies, and the exploration of other planets such as Venus^2^,^3^,^4^). Thermal logic devices exclusively employ heat (instead of electricity) to perform logic operations (i.e., calculations), hence they can be potentially powered by waste heat (~60% of the US energy consumption^5^). Thermal rectification-based logic devices (e.g., thermal diodes^6^,^7^,^8^,^9^, transistors^10^,^11^,^12^, memories^13^,^14^,^15^, and logic gates^16^,^17^) have been proposed theoretically and experimentally. Thermal diodes (i.e., rectifiers) received the most attention among the other thermal logic devices. This may be understood if we compare them to their electronic counterparts, which had a great impact on electronic logic systems (i.e., computers). Thermal diodes are devices that allow heat flow rates in a certain direction to be higher than in the opposite direction. Thermal rectification has been achieved using various techniques^18^, such as asymmetric nanostructures^19^,^20^, non-uniform mass loading^21^, and recently near-field thermal radiation (NFTR)^7^,^22^,^23^,^24^,^25^,^26^,^27^,^28^. However, the technologies proposed so far operate at cryogenic or room temperatures. In addition, the operation of these thermal rectifiers depends on temperature-dependent material properties, which limit their operating temperature range. Here we show experimentally—for the first time—the use of near-field thermal radiation (NFTR) to achieve thermal rectification at high temperatures, which can be used to build high temperature thermal diodes.

NFTR is a mode of transferring heat via thermal radiation between two surfaces, which occurs when the vacuum gap separating them becomes very small (i.e., comparable to the radiation wavelength)^29^. NFTR’s intensity has been shown to increase exponentially with decreasing separation gap^30^. The increased NFTR intensity results from the tunneling of the evanescent surface waves between the two surfaces as they are brought close enough to each other. In comparison to far-field thermal radiation, NFTR is therefore more suitable for heat transfer tailoring applications^31^ since its intensity can be controlled by carefully manipulating the separation gap. However, almost all proposed NFTR-based thermal rectification studies ignored this feature, rather utilized different techniques such as phase change in vanadium dioxide^7^,^23^,^28^, or temperature-dependent radiative properties^22^,^24^,^25^,^26^. This might be owing to the technical challenges to manipulate at the nanoscale vacuum gaps separating relatively large surface areas. Here in this research, we achieve thermal rectification between two temperature terminals by coupling their separation vacuum gap to their respective temperatures. This coupling allows for the radiative heat transfer rate between the two terminals (which depends on the gap size) to be a function of heat flow direction; this is the essence of thermal rectification. We have named this technique *NanoThermoMechanical Rectification* (NTMR).

## Results and Discussion

**Figure 1:**
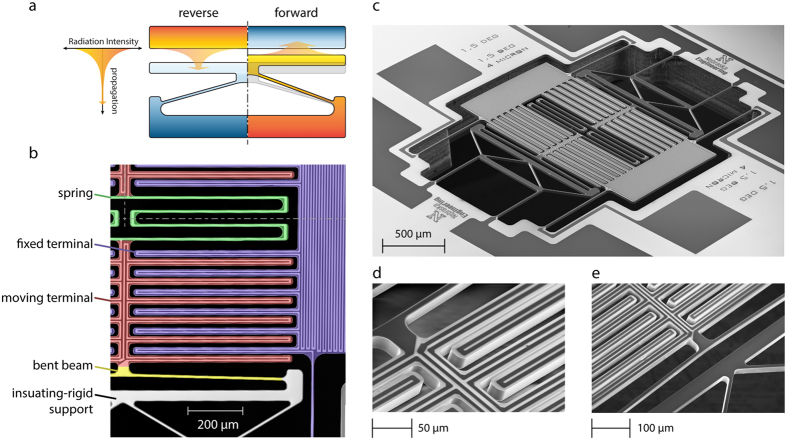
Near-field NanoThermoMechanical rectifier. (**a**) Near-field NanoThermoMechanical rectification concept. The rectifier is composed of a fixed terminal (at the top), a moving terminal (at the bottom), and a thermally-expandable structure (i.e., the v-shaped bent beam). The reverse and forward bias cases are represented in the left and the right halves of the sketch, respectively. The variable thickness arrow represents the decrease in thermal radiation intensity as radiation travels away from the heated surface. (**b**) False-color scanning electron micrograph of a quarter of the proof-of-concept microdevice, symmetric about the two perpendicular drawn symmetry lines. The microdevice incorporates 24 pairs of fixed and moving terminals in total, only 6 pairs are shown in (**b)**. (**c**) scanning electron micrograph of the proof-of-concept microdevice. (**d**,**e)** are zoomed-in views showing the connection of the moving terminal to the folded-beam spring and the bent beam, respectively.

The NanoThermoMechanical rectifier is conceptually composed of a fixed terminal (the upper part), a moving terminal (the lower part), and a thermally-expandable structure connected to the moving terminal, as shown in Fig. 1a. The principle of operation is illustrated in Fig. 1a: when the fixed terminal’s temperature is set high and the moving terminal’s temperature is set low (left half of Fig. 1a), then the expansion mechanism (the v-shaped bent-beam actuator) is not activated and the separation gap between the two terminals is unchanged (i.e., large). Since the NFTR decreases exponentially with the increase in the separation gap (as represented by the variable-thickness arrow), the larger gap corresponds to lower heat transfer rate. This case is referred to as reverse bias. Once the temperatures are switched, the moving terminal, which is now set to a higher temperature (right half of Fig. 1a), moves towards the fixed one. Consequently, the separation gap is decreased, which in turn increases the heat transfer rate by NFTR. This case is referred to as forward bias. The fabrication of a NanoThermoMechanical diode, such as the one sketched in Fig. 1a, can be achieved using conventional microfabrication techniques. However, measuring the heat transfer between the two terminals can pose a formidable challenge. This is due to the relatively low thermal radiation heat transfer rates in comparison to conduction heat transfer through the terminals’ supports, which has an effect on increasing the uncertainties in the heat transfer measurement.

To increase the accuracy of heat transfer measurements, we have designed and fabricated a proof-of-concept microdevice that employs 24 pairs of moving and fixed terminals sharing the same slim-isolating-supporting structure as shown in Fig. 1b–e. The microdevice was made out of a 20-μm-thick silicon substrate using standard microfabrication techniques. The microdevice’s main components are shown in Fig. 1b. In addition to the basic components of NanoThermoMechanical rectifier, we have included two thin-film platinum microheaters to manipulate and measure the temperatures of the two terminals independently, and to measure the heat transfer between them. The microdevice is symmetric about two perpendicular axes, as shown in Fig. 1b, causing the pairs of moving and fixed terminals to be divided into two groups. Each group of moving terminals is connected to a thermally-expandable structure (i.e., bent beam) from one side. Both groups are connected to a folded-beam spring from the remaining side to allow them to move freely in opposite directions. This arrangement was specifically employed to achieve parallelism among the fixed and moving terminals and to reduce thermal losses. Video recordings of the motion of the moving terminal are included in the Supplementary Information in Movie 1.

**Figure 2:**
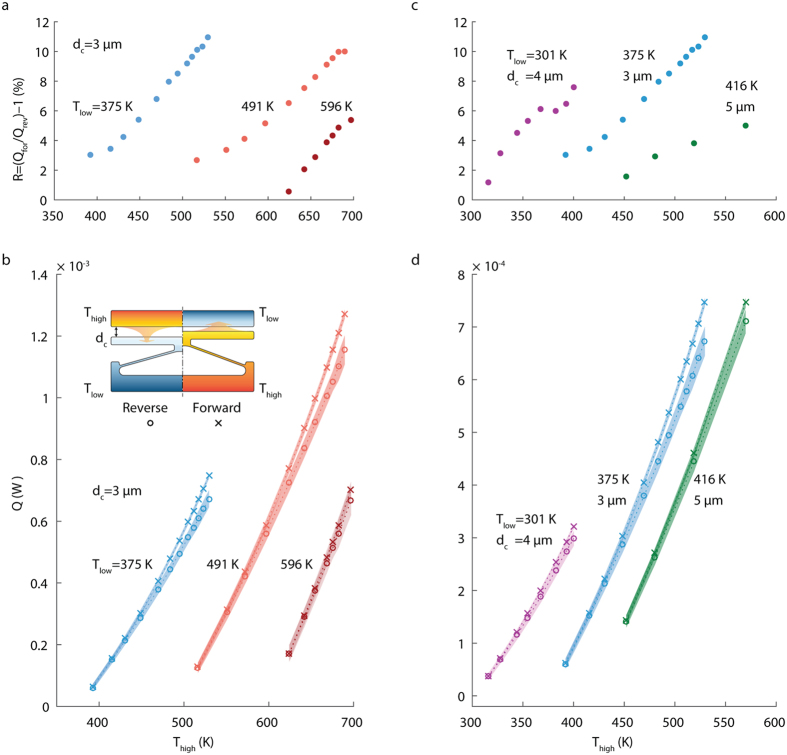
Thermal rectifier measured performance. (**a**) Thermal rectification (R = Q_for_/Q_rev_ − 1) versus T_high_ for a thermal rectifier device with initial separation gap (d_c_) of 3 μm. The thermal rectification is displayed for three different values of T_low_ (375, 491 and 569 K). The first data set was collected while chuck temperature was set to 350 K, and the other two data sets were collected at a chuck temperature of 450 K. (**b**) The heat transfer rate across rectifier terminals in forward and reverse directions used to calculate the thermal rectification in **a**. The inset in **b** shows the assignment of T_low_ and T_high_ in forward and reverse directions, and the initial separation gap d_c_. Uncertainties in the measured heat transfer rates are represented by the shading around measurement points. (**c**) shows thermal rectification versus T_high_ for three thermal rectifier devices with different initial separation gaps (3, 4 and 5 μm), at T_low_ of 375, 301 and 416 K, respectively. Chuck temperature was set to 350, 296, and 400 K, respectively. (**d)** represents the heat transfer rates across rectifier terminals in forward and reverse directions used to calculate the thermal rectification in **c**.

We have measured thermal rectification for three microdevices, all of them have the same bent-beam angle of inclination 3° to the horizontal, but they differ in the initial separation gap between the moving and the fixed terminals (3, 4 and 5 μm). In order to measure thermal rectification for a pair of low and high temperatures (T_low_, T_high_), we measured the heat transfer rate between the two terminals in forward (Q_for_) and reverse (Q_rev_) bias, and rectification can be estimated from the relationship R = (Q_for_ − Q_rev_)/Q_rev_. The forward bias scenario is achieved when T_low_ and T_high_ are assigned to fixed and moving terminals, respectively, while the reverse bias scenario is the opposite (as shown in the inset in Fig. 2b). Heat transfer measurements in the forward and reverse bias are plotted in Fig. 2b,d, and corresponding rectifications are shown in Fig. 2a,c for three different initial separation gaps. As shown in these plots, heat transfer rates in the forward bias are always higher than in the reverse one. As expected, measured thermal rectifications are shown to increase with the increasing T_high_, since the change in the separation gap is higher between the reverse and forward bias cases. The maximum thermal rectification achieved is 10.9% for T_low_ = 375 K and T_high_ = 530 K for the microdevice with a 3 μm initial separation gap. The microdevice has also shown thermal rectification at T_low_ as high as 596 K, with a value of 5.3%. This suggests that our technology can operate at elevated ambient temperatures as high as 600 K without cooling. In the current experiments, the maximum operating temperatures for the microdevice was limited by the stability of the platinum microheater, which showed unstable temperature-resistance relationship when heated above 800 K. Therefore, we believe that the microdevice can operate at higher temperatures with even higher thermal rectifications. Figure 2c,d compare the rectification for the three different gap size microdevices; it is evident that all microdevices show similar trends for heat transfer rate and thermal rectification, with thermal rectification increasing with decreasing initial separation gap, suggesting that the thermal rectification can be further augmented through design optimization.

In summary, we have demonstrated experimentally–for the first time in the literature–near-field NanoThermoMechanical rectification at high temperatures. Thermal rectification was achieved through the coupling between near-field heat transfer and the terminals’ separation gap, engineered to be a function of heat flow direction. Thermal rectifications were measured in a wide range of temperatures. Thermal rectification as high as 10.9% was achieved with T_low_ and T_high_ set at 375 and 530 K, respectively. Higher thermal rectifications can be achieved through further enhancement of the Near-field heat transfer using materials that supports stronger surface phonon or plasmon polaritons and by reducing the unrectified heat transfer between terminals. Thermal rectifications at temperatures as high as 600 K were measured, demonstrating for the first time thermal rectifications at such high temperatures. This invention opens the prospect for the development of thermal logic and thermal energy storage technologies of the future.

## Methods

### Device design

**Figure 3:**
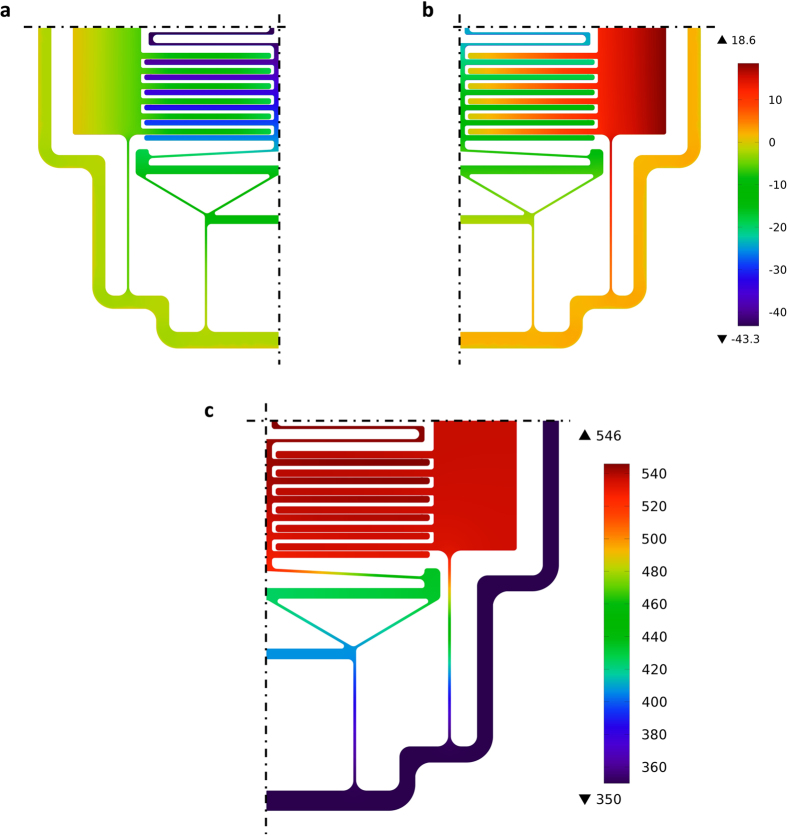
Results of finite element analysis for the proof-of-concept microdevice. (**a**,**b)** out-of-plane displacement for the bottom and top surfaces, respectively, unit on the scale is nanometers. (**c)** temperature distribution of the microdevice, unit on the scale is Kelvins.

To assess the feasibility (i.e., reduced conduction heat losses, heater temperature uniformity, and structural integrity) of the proposed design, we analyzed the proof-of-concept microdevice using finite element analysis via COMSOL Multiphysics^®^ (COMSOL, Inc., Burlington, MA, USA). The results of the numerical analysis are displayed in Fig. 3. In the simulation, heaters on fixed and moving terminals were powered using constant heat flux boundary conditions while the microdevice’s base temperature was set to 350 K. Temperature-dependent silicon properties were utilized in this analysis with the coefficient of thermal expansion and thermal conductivity adopted from refs 32 and 33, respectively. Figure 3a,b shows the microdevice’s out-of-plane displacements resulting from thermal stresses. It can be noted that the displacement of the top surface is positive (i.e., upwards); this is caused by the thermal expansion of the 20-μm-thick silicon device layer in the out of plane direction. This indicates that the out-of-plane deflection of the terminals is small and comparable to the thermal expansion of the silicon device layer. Figure 3c shows the temperature distribution of the microdevice. As can be seen from the figure, the temperatures of both terminals are relatively uniform with temperature range less than 6 K. The use of the slim supporting structures turned out to play a vital role in both reducing parasitic conduction losses and ensuring temperature uniformity of the terminals.

### Device fabrication

**Figure 4:**
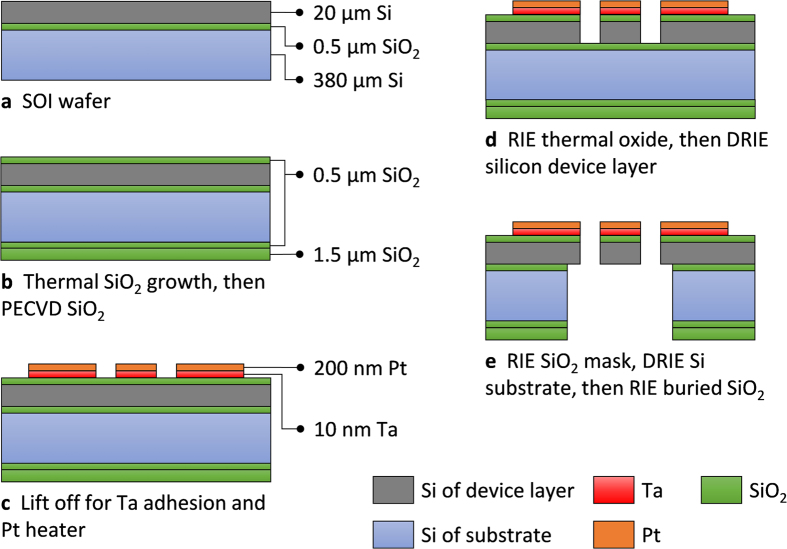
Fabrication steps of the Near-field NanoThermoMechanical rectifier (drawings are not to-scale). (**a**) Plain SOI wafer with a 20-μm thick device layer, and 0.5-μm buried silicon dioxide layer. (**b**) Thermal growth of 0.5-μm silicon dioxide layer, and plasma enhanced chemical vapor deposition of 1.5 μm of silicon dioxide. (**c**) Platinum microheater patterning with tantalum adhesion layer. (**d)** Reactive ion etching the (RIE) thermal silicon dioxide layer and then deep reactive ion etching (DRIE) of the silicon device layer to form the microdevice structure. (**e**) Etching of the backside of the substrate to release the microdevice structure.

The proof-of-concept microdevice was fabricated using in-cleanroom standard microfabrication techniques starting with a four-inches-diameter <100> silicon over insulator (SOI) wafer. The SOI wafer consists of a 380-μm thick handle silicon substrate, a 500-nm thick buried silicon dioxide layer, and a 20-μm thick boron-doped silicon device layer. Figure 4 shows the microfabrication process flow adopted to fabricate the current microdevices. Following a cleaning step of the wafers, a 0.5-μm thick silicon dioxide film was thermally grown by wet oxidation in a furnace at 1100 °C (Fig. 4b) on both sides of the wafer. This layer of silicon dioxide on the top side acts as an electrical insulator between the conductive silicon device layer and the subsequent electric heater. On the substrate’s backside, an additional 1.5 μm thick film of silicon dioxide was deposited via plasma enhanced chemical vapor deposition (PECVD) to serve as an etching mask in subsequent backside etch steps. After thermal oxidation, the microheater was formed on top of the device layer using lift-off as shown in Fig. 4c. In this step, a 10-nm thick tantalum (i.e., adhesion layer) and a 200-nm thick platinum (i.e., the electric heater) layers were sequentially deposited on top of a patterned LOR 5A photoresist using electron beam evaporation. Following the formation of the microheaters, the suspended structures of the NanoThermoMechanical rectifier (Fig. 4d) were formed through steps of reactive ion etching (to remove the 0.5-μm thick thermal silicon dioxide layer) and deep reactive ion etching of the silicon device layer. To release the final structures, backside etching (Fig. 4e) on the silicon dioxide, silicon handle wafer, and buried oxide were performed. All photolithography steps were done using projection lithography with 4:1 reduction and λ = 248 nm light source.

### Electrical characterization

Microdevice characterization and heat transfer measurements were performed inside a vacuum probe station at vacuum levels below 10^−5^ mbar, this eliminates convection and conduction heat transfer between terminals^34^. The two microheaters patterned on the fixed and the moving terminals were powered simultaneously and independently via two source-meter units (Keithley 2602 B) with a maximum voltage and current of 25 V/0.86 mA and 12 V/1.1 mA, respectively. The terminals’ temperatures were determined from knowing the electrical resistance of the microheaters through a careful temperature coefficient of resistance (TCR) calibration. TCR calibration was carried out by varying the temperature of the chuck (which holds the microdevice inside the vacuum chamber) from room temperature to 600 K and measuring the corresponding microheaters’ electrical resistances. Then, the resistance of each microheater was fitted to the corresponding temperature using a quadratic relationship^35^. To acquire stable TCR relationship, we annealed the heaters before the TCR calibration. The annealing was performed by setting the chuck temperature to 600 K, and supplying the maximum allowable current to the heaters while measuring their resistance. We cycled the current on and off ten times or more until the resistances became invariant, which indicates a stable TCR relationship. To decrease the errors associated with the two-probe measuring technique, the microheaters were designed to have high electrical resistances: 16000 and 6000 Ω at ambient temperatures for the fixed and moving terminal, respectively. The high resistance of the heaters relative to the electrical via (which was printed over the supports) ensured that over 95% of the energy supplied was dissipated at the terminals (i.e., heaters).

### Heat transfer measurement

**Figure 5:**
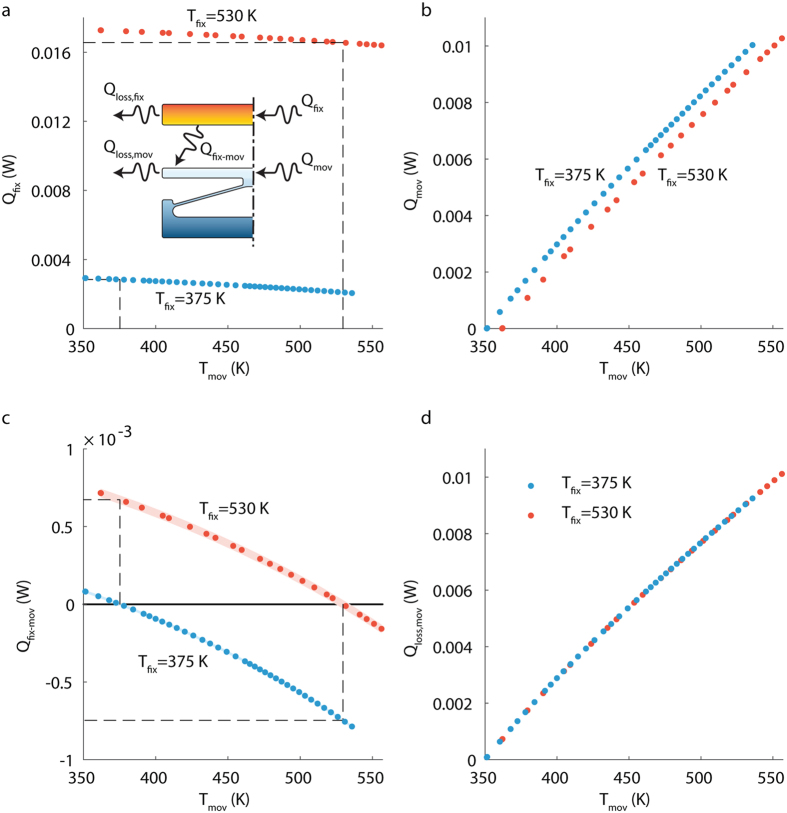
Measurement of heat transfer rate across terminals. (**a**) Measured heat dissipated in the fixed terminal (Q_fix_) versus temperature of the moving terminal (T_mov_). Each set of Q_fix_ and all other related measurements were conducted while the fixed terminal temperature (T_fix_) was kept at a constant value. The two data sets correspond to two temperature values for T_fix_: 375 and 530 K. (**b**) Measured heat dissipated in the moving terminal (Q_mov_) versus T_mov_, for two different T_fix_ values of 375 and 530 K. Uncertainties were not plotted in **a** and **b** since they were low, less than 0.16% and 0.29% for Q_fix_ and Q_mov_, respectively. (**c**) heat transfer from the fixed to the moving terminal (Q_fix-mov_) versus T_mov_ for the two different T_fix_ values. The dashed lines illustrate the heat transfer across terminals in forward and reverse directions for the set of temperature of 375 and 530 K. Uncertainties in the heat transfer rates are represented by the shaded area around the data points, with a maximum value of 2.74 × 10^−5^ W. (**d**) Heat losses from the moving terminal (Q_loss,mov_) versus T_mov_, for the two different T_fix_ values. Uncertainties were not plotted here as they were low, less than 3.89 × 10^−5^ W. Uncertainty was calculated based on the technique reported in ref. 36, more details in Supplementary Information.

Heat transfer measurements were conducted for each microdevice by keeping the fixed terminal’s microheater resistance constant (and accordingly its temperature), while sweeping the moving terminal’s temperature over the entire possible temperature range. Measured power dissipation from the fixed and the moving terminals’ microheaters are shown in Fig. 5a,b, respectively, with the chuck temperature set to 350 K. The device employed in this measurement has an initial separation gap of 3 μm and chevron angle of inclination of 3° degrees. The data sets shown here correspond to two temperature values for the fixed terminal: 375 and 530 K, however the same procedure applies to any variation of temperature set. The relationships among heat flow components are represented in the inset of Fig. 5a, and can be summarized with the following energy conservation equations:


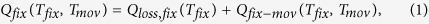



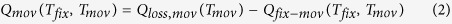


where terminals’ temperatures were assumed to be uniform (refer to Fig. 3c for temperature distribution calculated using finite element analysis). According to energy-balance and the second law of thermodynamics, heat transfer between the terminals ceases (i.e., Q_fix-mov_ = 0) when their temperatures are equal, and all power dissipated in the microheater of the fixed terminal must be lost to the surrounding environment by radiation and conduction through the supports (i.e., Q_loss,fix_ = Q_fix_). Using this knowledge, heat losses from the fixed terminal can be determined; they are graphically represented by the black dashed lines in Fig. 5a. Since the temperature of the fixed terminal was kept constant throughout each set of experiments, the term Q_loss,fix_ remained the same for the whole data set. Therefore, the heat transfer rate from the fixed to the moving terminals can be calculated from equation (1), the result of which is shown in Fig. 5c. The uncertainty in measuring the heat transfer rate is represented in Fig. 5c as a shading around the trend of the measurement points. The uncertainty was estimated based on the technique introduced by Robert J. Moffat^36^. The details of uncertainty analysis of our experimental procedure are listed in the Supplementary Information. Knowing Q_fix-mov_, heat losses from the moving terminal can be calculated from equation (2) and are displayed in Fig. 5d. As expected, heat losses from the moving terminal are independent of the fixed terminal’s temperature. Once the heat transfer rate for the two sets of fixed terminal’s temperatures (i.e., 375 and 530 K) are known for a range of moving terminal temperatures, the thermal rectification can be calculated as illustrated by the black dashed lines in Fig. 5c. We believe that no contact occurred between the fixed and moving terminals, since the heat transfer trend monotonically increase with the increase in the temperature of the moving terminal, without any kinks. We have tested the fabricated microdevice at higher values of current (corresponding to elevated terminal temperatures) to experience the contact between the fixed and moving terminals, and we were able to identify the contact visually as unusual movements of the terminals which drives them away from being parallel. We couldn’t measure either temperatures or heat transfer rates since the temperature-resistance relationship turn to be unstable at these elevated currents. We weren’t able to track and measure the separation distance between the fixed and moving terminals while performing the experiments for two reasons. The first reason is the heating caused by the microscope light, which interferes with the calculation of the heat transfer rate. The second reason is the limited resolution of the optical microscope attached to the vacuum probe station in our lab (~2 μm), which will indeed introduce large errors in estimating the separation distance.

## Additional Information

**How to cite this article**: Elzouka, M. and Ndao, S. High Temperature Near-Field NanoThermoMechanical Rectification. *Sci. Rep.* **7**, 44901; doi: 10.1038/srep44901 (2017).

**Publisher's note:** Springer Nature remains neutral with regard to jurisdictional claims in published maps and institutional affiliations.

## Supplementary Material

Supplementary Information

Supplementary video
