# Supplementary material for: High Temperature Near-Field NanoThermoMechanical Rectification
Source: Sci Rep. 2017 Mar 21;7:44901. doi: 10.1038/srep44901 (PMC5359666; doi:10.1038/srep44901)
Supplement: Supplementary Information [file srep44901-s1.pdf]

High Temperature Near-Field NanoThermoMechanical Rectification

Mahmoud Elzouka[1], and Sidy Ndao[1*]

[1]Department of Mechanical & Materials Engineering, University of Nebraska-Lincoln

Lincoln, Nebraska 68588, United States

Supplementary Information

*Correspondence to sndao2@unl.edu

Uncertainty analysis

In our experiment for measuring thermal rectification, we have the following uncertainties:

1- Uncertainty in electrical measurements for current, voltage, resistance and dissipated power (denoted by I, V, R, and P, respectively) for both fixed and moving terminals.
2- Uncertainty in chuck temperature measurement (denoted by T_{ch})
3- Uncertainty in the correlation between the coil resistance and its temperature (denoted by δTCR)
4- Uncertainty in estimating terminal temperature (denoted by δT)
5- Uncertainty in the correlation between $\Delta T = T_{fix} - T_{mov}$ and Q_h (to get the $Q_{loss,fix}$)
6- Uncertainty in Correlation between T_{mov} and $Q_{fix-mov}$ (to estimate uncertainty in $Q_{fix-mov}$)
7- Uncertainty in Correlation between T_{mov} and $Q_{loss,mov}$ (to estimate uncertainty in $Q_{loss,mov}$)

The analysis of the aforementioned uncertainties is explained in the following sections:

1- Uncertainty in electrical measurements for current, voltage, resistance and dissipated power in terminals

Electrical measurements were performed using Keithley SourceMeter2602 B. The uncertainty in measured voltage and measured current was adopted from the datasheet document '*Model 2601A/2602A System SourceMeter® Specifications*' [1]

a. Error in measured current δI

Current range	Uncertainty δI
$\leq 100\ uA$	$0.02\% \times I + 25\ nA$
$\leq 1\ mA$	$0.02\% \times I + 200\ nA$
$\leq 10\ mA$	$0.02\% \times I + 2.5\ \mu A$

b. Error in measured voltage

Voltage range	Uncertainty δV
$\leq 1\ V$	$0.015\% \times V + 200\ \mu V$
$\leq 6\ V$	$0.015\% \times V + 1\ mV$
$\leq 40\ V$	$0.015\% \times V + 8\ mV$

The uncertainty in the calculated resistance and power were calculated using the technique published in [2]:

c. Uncertainty in calculated electrical resistance

From the resistance formula $R = \frac{V}{I}, \frac{\partial R}{\partial V} = \frac{1}{I}, \frac{\partial R}{\partial I} = -\frac{V}{I^2}$, we can estimate the uncertainty in resistance δR;

$$\delta R = \sqrt{\left(\frac{\partial R}{\partial V}\delta V\right)^2 + \left(\frac{\partial R}{\partial I}\delta I\right)^2} = \sqrt{\left(\frac{1}{I}\delta V\right)^2 + \left(-\frac{V}{I^2}\delta I\right)^2}$$

d. <u>Uncertainty in calculated electrical power dissipation</u>

From the electrical power dissipation $P = IV, \frac{\partial P}{\partial V} = I, \frac{\partial P}{\partial I} = V$, the power dissipated can be estimated from;

$$\delta P = \sqrt{\left(\frac{\partial P}{\partial V}\delta V\right)^2 + \left(\frac{\partial P}{\partial I}\delta I\right)^2} = \sqrt{(I\delta V)^2 + (V\delta I)^2}$$

2- Uncertainty in chuck temperature measurement (denoted by δT_{ch})

Chuck temperature measurement were performed using Lake Shore temperature controller (335 series). We have used a resistance temperature detector (RTD) made of platinum, with a positive temperature coefficient (PTC). The temperature measurement error was adopted from the user's manual[3], which was 62 mK for a temperature range below 300 K, and 106 mK otherwise.

3- Uncertainty in the correlation between the coil resistance and its temperature (denoted by δTCR)

The temperature coefficient of resistance (TCR) is calculated from the experimental relationship between the terminal resistance and its corresponding temperature (i.e., chuck temperature). The TCR relation is found by regression analysis; by fitting the experimental data points corresponding to terminal resistance and its corresponding temperature (i.e., chuck temperature) to a quadratic relationship. The uncertainty in the TCR at each point (δTCR) is assumed to be the maximum of the two values; δT, and $\delta R \times dT/dR$, as illustrated in Supplementary Fig. S1 (assuming that x and y are corresponding to R and T, respectively). dT/dR is determined by the TCR relationship (i.e., the slope of the fitting curve).

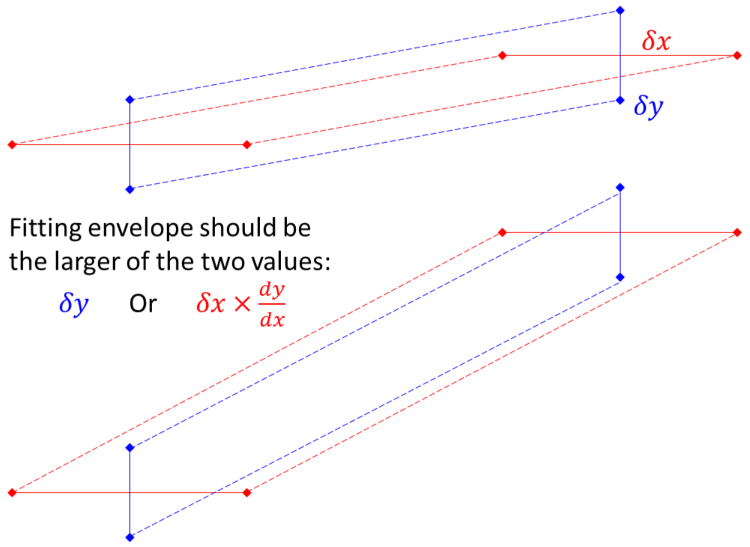

Supplementary Figure S1

4- Uncertainty in estimating terminal temperature (denoted by δT)

During heat transfer experiments, the terminal temperature is estimated from resistance measurement. The uncertainty of estimated temperature can be evaluated from the relationship $\delta T = \delta R \times \frac{dT}{dR} + \delta TCR$, which can be inferred geometrically from Supplementary Fig. S2. $\frac{dT}{dR}$ is evaluated from the TCR relationship. For example, if the fitting has the form $T = aR^2 + bR + c$, then $\frac{dT}{dR} = 2aR + b$.

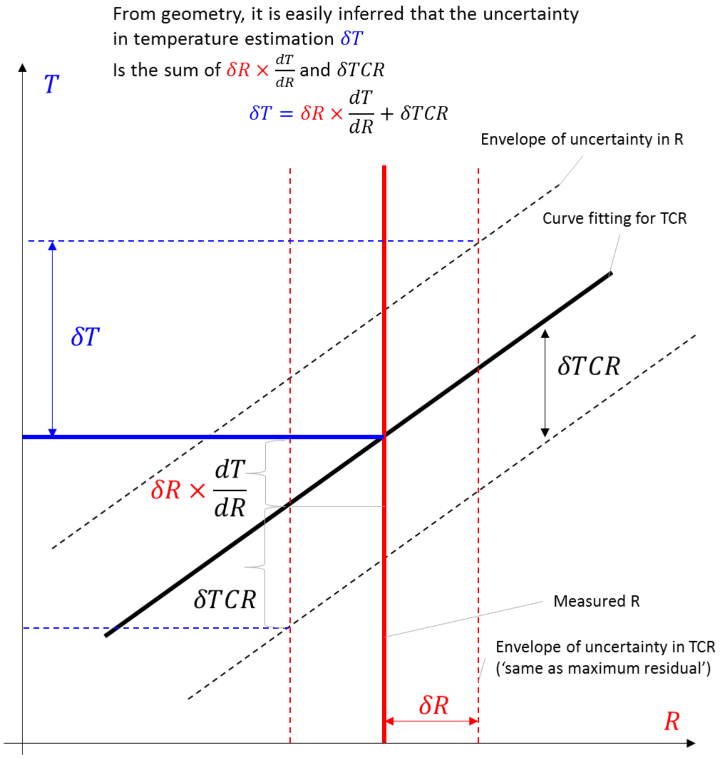

Supplementary Figure S2

5- Uncertainty in the correlation between $\Delta T = T_{fix} - T_{mov}$ and Q_h (to get the $Q_{loss,fix}$)

a. Uncertainty in $\Delta T = T_{fix} - T_{mov} = T_{fix-mov}$
The uncertainty in the temperature difference $\delta T_{fix-mov}$ is evaluated with the method listed in [2];

$$\delta T_{fix-mov} = \sqrt{\left(\frac{\partial T_{fix-mov}}{\partial T_{fix}}\delta T_{fix}\right)^2 + \left(\frac{\partial T_{fix-mov}}{\partial T_{mov}}\delta T_{mov}\right)^2} = \sqrt{\left(\delta T_{fix}\right)^2 + (-\delta T_{mov})^2}$$
$$= \sqrt{\delta T_{fix}{}^2 + \delta T_{mov}{}^2}$$

b. Uncertainty in fitting the relation between $T_{fix-mov}$ and Q_{fix}

The uncertainty in the fitting relationship between $T_{fix-mov}$ and Q_{fix} can be evaluated using Supplementary Fig. S1; and it is the maximum of δQ_{mov} and $\delta T_{fix-mov} \times \frac{dQ_{mov}}{dT_{fix-mov}}$

c. Uncertainty in $Q_{loss,fix,0}$

It is the uncertainty in fitting the relation between $T_{fix-mov}$ and Q_{fix}, but evaluated at $T_{fix-mov} = 0$.

6- Uncertainty in Correlation between T_{mov} and $Q_{fix-mov}$ (to estimate uncertainty in $Q_{fix-mov}$)

$Q_{fix-mov}$ is calculated from $Q_{fix-mov} = Q_{fix} - Q_{loss,fix,0}$. Accordingly, the uncertainty in the heat transfer value $\delta Q_{fix-mov}$ is evaluated from;

$$\delta Q_{fix-mov} = \sqrt{\left(\frac{\partial Q_{fix-mov}}{\partial Q_{fix}} \delta Q_h\right)^2 + \left(\frac{\partial Q_{fix-mov}}{\partial Q_{loss,fix,0}} \delta Q_{loss,fix,0}\right)^2}$$

$$= \sqrt{\left(\delta Q_{fix}\right)^2 + \left(-\delta Q_{loss,fix,0}\right)^2} = \sqrt{\delta Q_{fix}{}^2 + \delta Q_{loss,fix,0}{}^2}$$

7- Uncertainty in Correlation between T_{mov} and $Q_{loss,mov}$ (to estimate uncertainty in $Q_{loss,mov}$)

$Q_{loss,mov}$ is calculated from $Q_{loss,mov} = Q_{fix} + Q_{mov} - Q_{loss,fix,0}$. Accordingly, the uncertainty in the heat losses from the moving terminal $\delta Q_{loss,mov}$ is evaluated from;

$$\delta Q_{lc} = \sqrt{\left(\frac{\partial Q_{lc}}{\partial Q_h} \delta Q_h\right)^2 + \left(\frac{\partial Q_{lc}}{\partial Q_c} \delta Q_c\right)^2 + \left(\frac{\partial Q_{lc}}{\partial Q_{lh0}} \delta Q_{lh0}\right)^2} = \sqrt{\delta Q_h{}^2 + \delta Q_c{}^2 + \delta Q_{lh0}{}^2}$$

References

1. Keithley Instruments, I. System SourceMeter® Specifications Model 2601A/2602A. (2011).
2. Moffat, R. J. Describing the uncertainties in experimental results. *Exp. Therm. Fluid Sci.* **1,** 3–17 (1988).
3. Lake Shore Cryotronics, I. User's Manual Model 335 Temperature Controller. (2014).
